# Dental Specialties

**Published:** 1865-02

**Authors:** 


### Editorial.

### DENTAL SPECIALTIES.

THE subject of division and subdivision of medical practice is one that has received considerable attention, at least practically, and some discussion.

There are divisions of medical practice, that men have always been disposed to regard favorably and practice upon.

It is a question perhaps not so easily determined, how far this division of practice shall extend; all will agree, however, that it should go so far as it would operate for the welfare of humanity, and the more effectual relief from disease and suffering. But it is equally true, that there are certain fundamental principles, and branches that should be thoroughly understood by all who attempt in any particular to treat disease. It matters not to what part of the human system attention is directed, pathological conditions will be found that are common to most, if not all other organs, or parts of the body, and so far as there is a full comprehension of such conditions in one part, there is of all others. It is also equally true, that every organ has some pathological condition and circumstances peculiar to itself; and in order that these may be the more thoroughly investigated, understood and treated, special attention should be given to them; hence, the importance and necessity of specialties in medical practice.

The importance and efficiency of dentistry as a specialty of Medical practice, is pretty fully recognized and acknowledged by all, though it has not long been so. And now, a question of increasing importance is, is not dental practice susceptible o further division, to the advantage of all concerned; and if so, to what extent?

Most operators acknowledge the fact, that a separation of the mechanical, and operative departments will be advantageous to all, where it is at all practicable, some regarding it so only in large cities, or where there are a considerable number of dentists in the same vicinity. This we do not think at all necessary. In the village or town that will sustain two dentists, such an arrangement may be profitably adopted, greatly to the benefit of all concerned, the one taking the operative, and the other the mechanical department, all things would go well, each one concentrating himself upon his own department, would attain greater efficiency than if he was cumbered by both branches; the work of both departments would be far better done, and more efficient, in consequence of such division.

Under the present arrangement the construction of artificial dentures is entrusted to the hands of beginners, and incompetent persons, the principal confining himself chiefly to the operative department.

The mechanical dentist, who should assume that department, would have a pride in having every thing done in the most perfect manner. Dentists should consult their tastes and inclination in the selection of their departments. Some are good mechanical dentists who would never make good operators upon the natural teeth; others operate well upon the natural teeth who should never attempt the construction of an artificial set.

By such an arrangement each would be interested in sustaining the other. As dental practice at present exists, the emolument derivable from the two departments is very nearly equal, then each one would be interested in sustaining the good name and reputation of the other, which, under the present circumstances, oftentimes is far otherwise.

Again, the practice of one department. in a very marked degree, unfits the dentist for the proper performance of the other. We have a few things still to say upon this subject.

